# Validation of a Deep-Learning Coregistration Framework for Long–Axial-Field-of-View PET/CT Using Low-Radiation-Exposure Protocols Across Various Tracers

**DOI:** 10.2967/jnumed.125.270420

**Published:** 2026-05

**Authors:** Zekai Li, Laura Providência, Philipp Mohr, Samaneh Mostafapour, Mostafa Roya, T. Samara Martinez-Lucio, Joost F. Somsen, Goudje L. van Leeuwen, Giordana Salvi De Souza, Joyce van Sluis, Andor W.J.M. Glaudemans, Rudi A.J.O. Dierckx, Jean-Paul P.M. de Vries, Gert Luurtsema, Riemer H.J.A. Slart, Adrienne H. Brouwers, Paul Schleyer, Maurizio Conti, Adriaan A. Lammertsma, Joshua Schaefferkoetter, Charalampos Tsoumpas

**Affiliations:** 1 Department of Nuclear Medicine and Molecular Imaging, University of Groningen, University Medical Center Groningen, Groningen, The Netherlands;; 2 Department of Surgery, Division of Vascular Surgery, University of Groningen, University Medical Center Groningen, Groningen, The Netherlands;; 3 Department of Biomedical Photonic Imaging, Faculty of Science and Technology, University of Twente, Enschede, The Netherlands; and; 4 Siemens Medical Solutions USA, Knoxville, Tennessee

**Keywords:** artificial intelligence, attenuation correction, registration, positron emission tomography

## Abstract

The advent of long–axial-field-of-view (LAFOV) PET/CT systems has significantly improved whole-body imaging by providing higher sensitivity and extended torso coverage. However, PET/CT potential misalignment remains a challenge, potentially introducing artifacts and quantification errors. Moreover, PET protocols with reduced scanning duration and dose, as well as the use of ultra-low-dose CT (ULD-CT), are increasingly relevant in clinical practice and research. This study aimed to evaluate the robustness and generalizability of a deep-learning coregistration framework for PET/CT alignment using low-dose PET and ULD-CT across various tracers with a LAFOV system. **Methods:** In total, 63 scans with 4 different tracers (^89^Zr-trastuzumab, ^15^O-H_2_O, ^18^F-MC225, and ^18^F-FDG) were included to assess PET and CT alignment improvements. Further evaluation was performed by comparing CT scans coregistered to the original PET images (rCT) with those coregistered to low-count (50%, 25%, and 12.5% of original counts) PET images (LC-rCT). Dice similarity coefficient, Jaccard similarity coefficient, Hausdorff distance, and average surface distance were used as evaluation metrics. Furthermore, ULD-CT coregistered to PET (rULD-CT) was compared with low-dose CT coregistered to the same PET (rLD-CT) using the same metrics. PET accuracy was evaluated by calculating SUVs. **Results:** The robustness of the deep-learning coregistration framework was demonstrated in reduced PET counts and ULD-CT scenarios across tracers. All metrics indicated robust performance when comparing LC-rCT with rCT and rULD-CT with rLD-CT. Consistent SUVs across varying PET counts and ULD-CT conditions further validated the quantitative accuracy of the approach. **Conclusion:** This work highlights that neither the use of low-dose PET protocols nor ULD-CT compromise the performance of this coregistration framework across 4 tracers. For LAFOV PET, these findings support the feasibility of such a framework in imaging protocols with reduced radiation exposure.

## Visual Abstract

**Figure:**
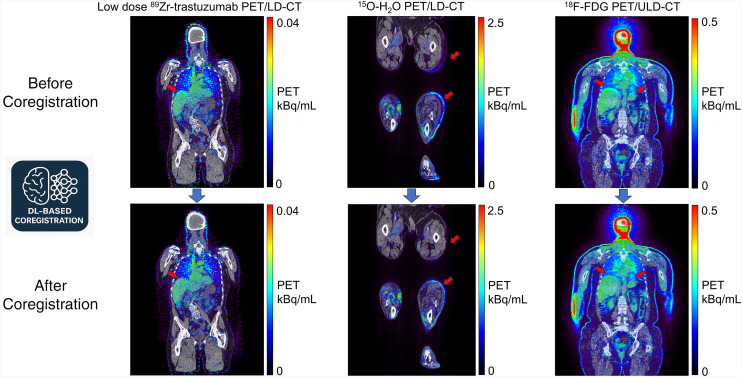

PET/CT is widely used in both clinical and research settings, driven by its ability to provide quantitative molecular imaging for a variety of applications. The addition of CT, most commonly performed as low-dose CT (LD-CT) (1), is primarily intended for generating attenuation correction maps to account for the scattering and attenuation of photon emissions and provide anatomic localization. Attenuation correction, which is essential for accurate PET quantification, is typically performed using attenuation correction CT (AC-CT) acquired immediately before or after the PET scan, assuming minimal patient movement between scans to ensure good alignment between PET and CT images. Voluntary motion is typically addressed by asking the subject to minimize movement or by using physical constraints (2). Involuntary motion, such as respiratory and cardiac movements, is impossible to constrain. Mismatches between PET and AC-CT introduce blurring and other artifacts that degrade image quality and spatial resolution (3–6).

Brain studies are easier to handle because the skull’s rigidity allows for the use of rigid-body registration techniques (7). In contrast, the thoracic and abdominal regions require more complex registration methods because of the presence of soft tissues that are subject to respiratory and cardiac motion. Both rigid and elastic registration algorithms have been developed for these regions (8,9). Subsequent work on whole-body PET/CT registration has typically combined affine and elastic transformations (10–12), which can be computationally intensive. Deep learning offers promising potential for whole-body PET/CT registration (13,14). Schaefferkoetter et al. (15) developed a deep-learning method for whole-body PET/CT, demonstrating its applicability across ^18^F-FDG, ^68^Ga-PSMA, and ^68^Ga-DOTATOC datasets. This method was further validated using ^64^Cu-DOTATATE datasets (16–18).

The advent of long–axial-field-of-view (LAFOV) PET/CT enabled imaging of larger portions of the body in a single bed position using lower amounts of administered tracer (19). In addition, ultra-low-dose CT (ULD-CT) allows for radiation dose reductions of over 90% at the expense of image quality (20). Combined with the growing number of tracers, these developments underscore the need for robust and generalizable whole-body PET/CT coregistration solutions (21,22).

The aim of this study was to evaluate the robustness and generalizability of the deep-learning PET and CT coregistration framework (15) in 3 previously unexplored conditions: 4 tracers, different body regions, and a LAFOV system; low PET statistics scenarios simulating lower-dose or reduced acquisition duration; and ULD-CT. These 3 aspects are critical for determining the feasibility of implementing this framework across different protocols.

## MATERIALS AND METHODS

### Image Registration and Reconstruction

A convolutional neural network–based framework has been developed and trained to perform registration between PET and CT images (15). Details of this network architecture and training procedure are provided in the supplemental materials, available at http://jnm.snmjournals.org. In the first step of the framework, raw PET data are reconstructed into non–attenuation-corrected (NAC)–PET images, which serve as input for registration. The second step performs NAC-PET and CT coregistration using a convolutional neural network architecture that incorporates a spatial transformer. This includes a U-Net–like feature extractor, which generates feature maps, and a displacement vector field regressor, which computes the 3-dimensional displacement field needed for alignment. The computed displacement field is then used to resample the CT volume, aligning it with the NAC-PET image. The resulting coregistered CT (rCT) image, along with the raw PET data, are used to reconstruct the PET/CT aligned attenuation-corrected PET (rPET) image. The entire process was automated using command line scripts interfaced with investigational reconstruction software (e7tools) provided by Siemens Healthineers. Both rPET and rCT images could then be uploaded to clinical imaging software for review.

All static PET images in this study, including NAC-PET, rPET, and images reconstructed using the original AC-CT, were reconstructed using 3-dimensional ordered-subset expectation maximization (5 subsets, 4 iterations) with point-spread function and time of flight. The matrix size was set to 220 × 220 × 645 (voxel size, 3.3 × 3.3 × 1.645 mm^3^) using vendor-suggested settings.

### Data and Study Participants

This study evaluated 63 scans obtained using the Biograph Vision Quadra PET/CT (Siemens Healthineers), a system with a 106-cm axial field-of-view (23). The central ethics review board for studies that fall outside the scope of the Medical Research Involving Human Subjects Act (CTc-UMCG) approved the conduct of the study (waivers METc2020/554, METc2022/426, CTc21725, and CTc10946). Subjects were injected with 1 of 4 tracers: ^15^O-H_2_O, ^18^F-MC225, ^89^Zr-trastuzumab, or ^18^F-FDG. This study retrospectively used previously published datasets without any PET/CT coregistration (24–27). Beyond these datasets, 4 of the 6 ^15^O-H_2_O torso scans were performed on a single volunteer to explore physiologic stressors as alternatives to adenosine-induced vasodilation in myocardial perfusion imaging. The PET/CT protocol included image acquisitions during rest, breath holding, Valsalva maneuvers, and adenosine stress. The primary aim was to assess myocardial blood flow and coronary flow reserve responses to these physiologic stressors, evaluating their feasibility as nonpharmacologic alternatives for patients with contraindications to adenosine. Twenty-seven ^18^F-FDG scans included an LD-CT performed before PET acquisition as part of standard care, followed by an ULD-CT after PET acquisition. In addition, 10 ^18^F-FDG scans included 15-s breath-hold PET acquisitions after CT. Study participants’ characteristics, scanning protocols, and injected activities are detailed in Table 1.

**TABLE 1.** Scan Protocols Using Different Tracers

Tracer	No. scans	Body region	PET acquisition time (s)	Injected activity	CT settings	
					kVp	mAs
^15^O-H_2_O	6	Head to upper legs	370 or 610	200 or 500 MBq	100–140	19–34
^15^O-H_2_O	2	Feet to abdomen	600	400 MBq	100–120	5
^18^F-MC225	10	Head to upper legs	3,610	200–360 MBq	100–140	10–30
^89^Zr-trastuzumab	8	Head to upper legs	1,800*	37 MBq	100–140	5
^18^F-FDG	27	Head to upper legs	600	2 MBq/kg	LD-CT, 100–120; ULD-CT, 100^†^	LD-CT12–30; ULD-CT: 6
^18^F-FDG	10	Head to upper legs	15^‡^	2–3 MBq/kg	100–120	17–21

* Day 4 after injection.

† With tin filtration.

‡ Breath-hold scan.

### Evaluation

#### PET/CT Misalignment Improvement

Qualitative comparisons were made between the original AC-CT PET and rPET images, focusing on the improvement of PET/CT alignment.

#### Reduced PET Counts

This test assessed the framework’s stability under lower count conditions, with the goal of exploring its potential for reduced dose or scan duration across tracers with distinct biodistributions. To this end, a fraction of the events in the raw data were randomly removed using the e7tools, where the total PET counts were progressively reduced to 50%, 25%, and 12.5% of the original counts. Next, the coregistration framework was applied to generate the rCT using the same CT in combination with PET images with reduced counts.

To assess the robustness of the CT registration to the low-count PET (LC-rCT), the Multiple-Organ Objective Segmentation 3.0 software (28) was applied to both LC-rCT and rCT images, and the resulting segmentations were compared. The segments included in the torso scans were liver, upper left lobe (lung), lower left lobe (lung), upper right lobe (lung), middle right lobe (lung), lower right lobe (lung), and spleen. To evaluate the similarity of segmented LC-rCT and rCT, the Dice similarity coefficient (DSC), Jaccard similarity coefficient (JSC), Hausdorff distance (HD), and average surface distance (AVD) were used across different tracers and segments, considering factors such as overlap, boundary delineation, and spatial accuracy (29).

After CT segmentation, the CT-derived regions were used to map the CT segmentations to the corresponding PET images and to obtain the SUV_mean_ for each segmented region. Bland–Altman analysis was used to compare the SUV_mean_ for each region between the PET images reconstructed with the rCT obtained from reduced count statistics and the PET images reconstructed with the rCT obtained from the original counts data.

#### ULD-CT

The coregistration framework was expected to reduce discrepancies between LD-CT and ULD-CT using the same PET data as the coregistration target, since both CT scans were acquired during the same PET session.

For this analysis, torso segmentations were performed using Multiple-Organ Objective Segmentation 3.0 software on LD-CT, ULD-CT, coregistered LD-CT (rLD-CT), and coregistered ULD-CT (rULD-CT). The DSC, JSC, HD, and AVD were calculated between rLD-CT and rULD-CT and compared with those obtained between the original LD-CT and ULD-CT to evaluate the effectiveness of the coregistration process. Differences in all metrics were assessed using Wilcoxon signed rank test, and a *P* value of less than 0.05 was considered statistically significant.

The SUV_mean_ of PET images reconstructed with LD-CT, ULD-CT, rLD-CT, and rULD-CT was calculated for each segmented PET region using their respective CT segmentations. Bland–Altman analysis was applied to evaluate the consistency of PET quantification between coregistered and noncoregistered CTs, providing a direct measure of the reliability of PET measurements using 2 CT dose levels.

## RESULTS

### PET/CT Misalignment Improvement

**FIGURE 1.**
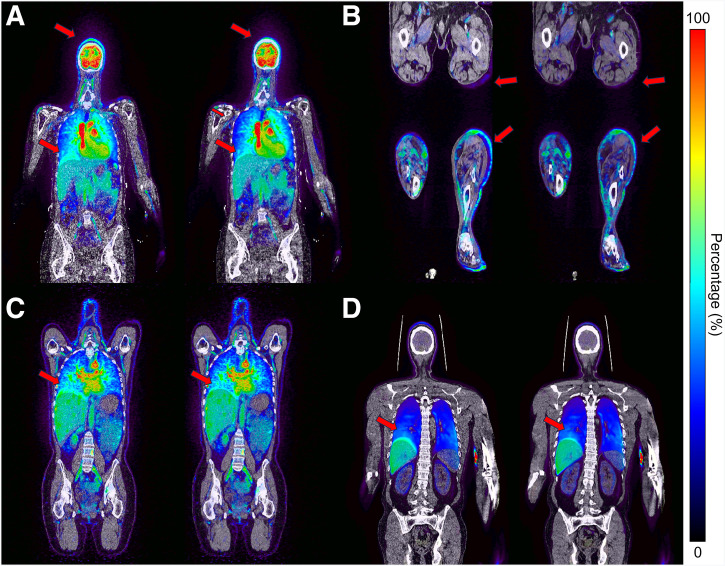
(A) ^15^O-H_2_O torso, (B) ^15^O-H_2_O legs, (C) ^89^Zr-trastuzumab torso, and (D) ^18^F-MC225 torso fused NAC-PET images with original AC-CT (left) and coregistered CT (right). Reduced mismatch between PET and CT indicated by red arrows.

Misalignments between NAC-PET and original CT images were observed in various cases, as in Figure 1. After applying CT to PET coregistration, improved alignment between PET and CT was observed for all tracers.

**FIGURE 2.**
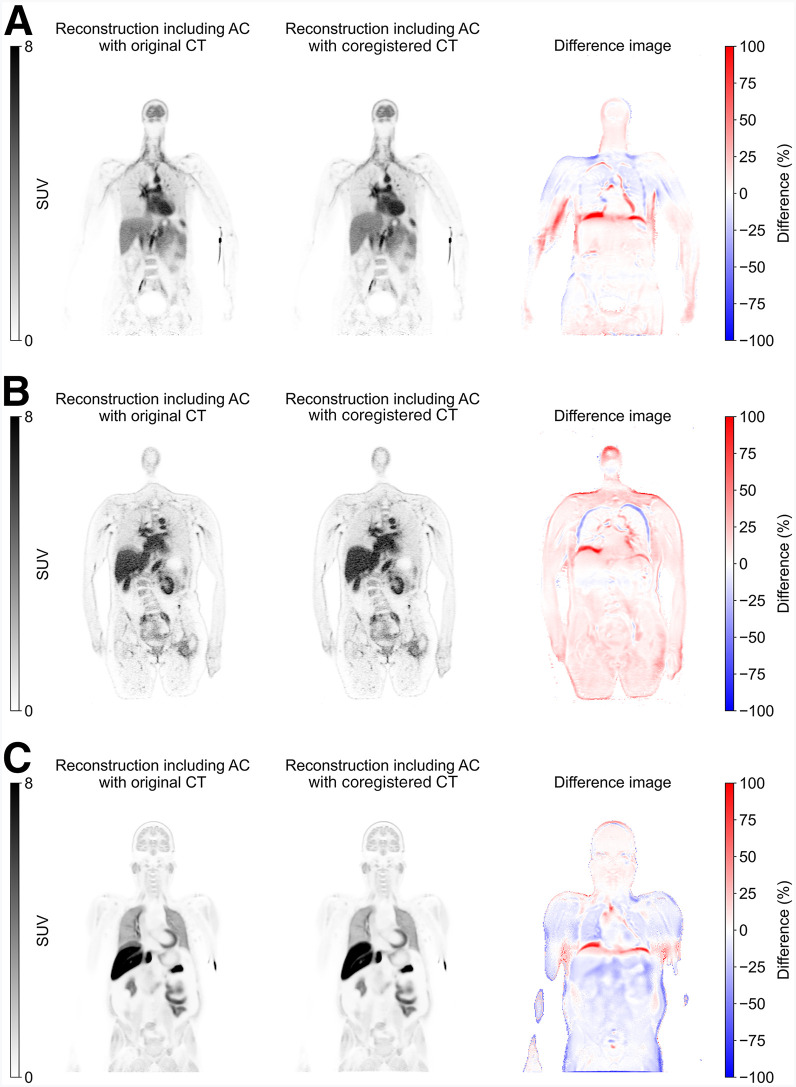
(A) ^15^O-H_2_O torso, (B) ^89^Zr-trastuzumab torso, and (C) ^18^F-MC225 torso PET images reconstructed using original AC-CT (left) and coregistered CT (middle), alongside relative difference maps (right). AC = attenuation correction.

Respiratory-induced artifacts, such as the banana-shaped artifact, typically occur when CT is acquired during deep inspiration compared with the average respiratory cycle, resulting in considerable misalignment between CT and PET images. These artifacts were notably reduced after coregistration (Fig. 2) and produced a more uniform and anatomically consistent tracer distribution within the liver (Supplemental Fig. 1).

**FIGURE 3.**
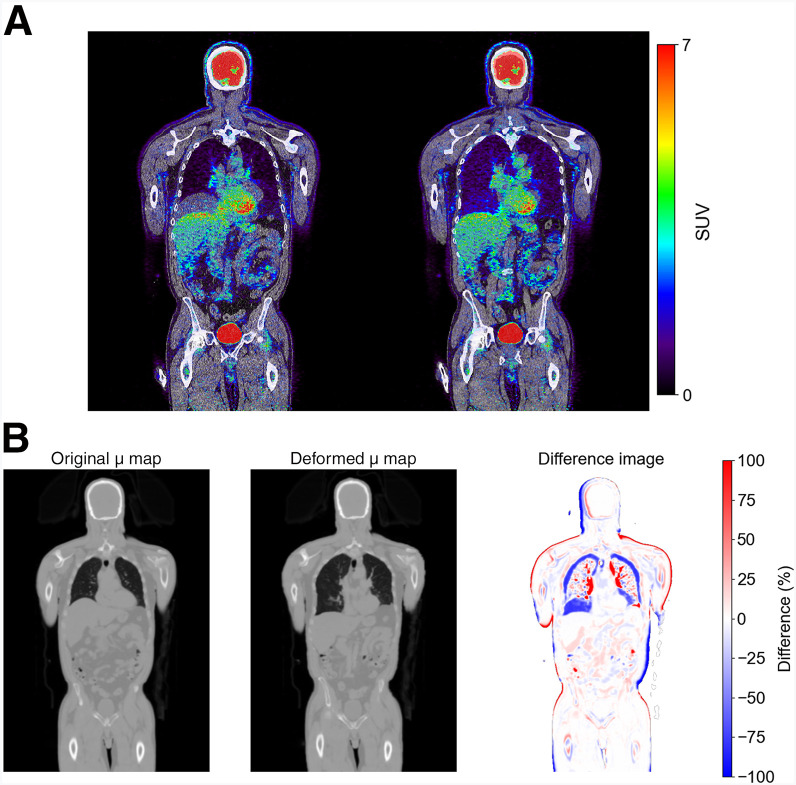
(A) Fused ^18^F-FDG breath-hold PET/CT images reconstructed using original AC-CT (left) and coregistered CT (right). (B) Comparison of μ map with deformed μ map, alongside difference image.

For the ^18^F-FDG deep-breath-hold scans (Fig. 3), extreme banana-shaped artifacts were observed when comparing the 15-s breath-hold PET with the CT image. These discrepancies were primarily caused by the increased lung volume because of the inhaled air during the deep-breath-hold (30). After applying the coregistration framework, mismatches at the diaphragm were significantly reduced. Furthermore, comparing the μ maps revealed notable differences between the μ map generated from the original AC-CT (original μ map) and that from the rCT (deformed μ map). Specifically, at the diaphragm, the values in the original μ map decreased significantly after coregistration. This change reflected a correction of the misclassification of a part of the lungs as the liver dome in the original μ map, underscoring the effectiveness of the coregistration framework in refining attenuation correction.

### Reduced PET Counts

**FIGURE 4.**
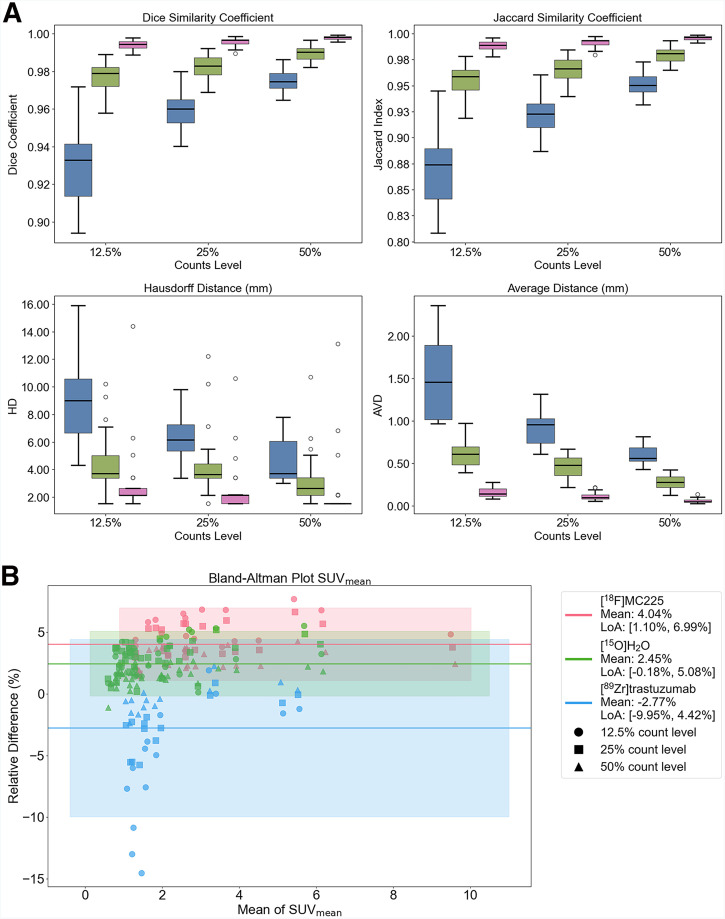
(A) Box plots comparing DSC, JSC, HD, and AVD among ^89^Zr-trastuzumab (blue), ^15^O-H_2_O (green), and ^18^F-MC225 (red) for 12.5%, 25%, and 50% count levels (outliers are depicted by circles). (B) Bland–Altman plots showing SUV_mean_ between reduced-counts groups (12.5%, 25%, 50%) and original-count groups for ^89^Zr-trastuzumab (blue), ^15^O-H_2_O (green), and ^18^F-MC225 (red).

Several reduced-count scenarios with notable PET/CT misalignments (5 ^15^O-H_2_O torso scans, 2 ^89^Zr-trastuzumab torso scans, 3 ^18^F-MC225 torso scans, and 1 ^15^O-H_2_O lower-leg scan) were used to evaluate the framework’s robustness. Breath-hold patients were excluded because of their short original scan time (15 s). High DSC and JSC values were observed for all tracers, indicating good overlap between LC-rCT and rCT (Fig. 4A). ^18^F-MC225 consistently achieved the highest median DSC (>0.99) and JSC (>0.98) across all count levels, remaining robust at 12.5% counts. ^15^O-H_2_O showed slightly lower values (DSC > 0.97, JSC > 0.94), whereas ^89^Zr-trastuzumab exhibited the lowest medians and highest interquartile ranges, particularly at 12.5% counts (median DSC < 0.94; median JSC < 0.87). Boundary delineation metrics (HD and AVD) reflected similar trends. ^18^F-MC225 had the lowest median HD and AVD at all count levels, indicating superior boundary accuracy, whereas ^15^O-H_2_O had slightly higher values. ^89^Zr-trastuzumab showed the highest median and highest IQR of HD and AVD values at 12.5% counts, but improvements were evident at higher count levels.

All organ segments showed high DSC and JSC and low HD and AVD (Supplemental Table 1), indicating good overlap between LC-rCT and rCT, which remained consistent across different segmented regions.

Results of the Bland–Altman plots for SUV_mean_ across tracers and count levels are presented in Figure 4B. All tracers demonstrated good consistency in SUV_mean_ quantification across all count levels, with mean relative differences below 5%. Among the tracers, ^15^O-H_2_O and ^18^F-MC225 exhibited robust performance across all count levels, characterized by narrow limits of agreement (LoA), indicating high reliability even at reduced counts. ^89^Zr-trastuzumab displayed the widest LoA [−9.95% to 4.42%] and the highest variability, particularly at the 12.5% lowest count level, where the spread of data points was notably wider. However, the variability in SUV_mean_ decreased progressively from 25% to 50% count levels for all tracers, with a marked improvement in the accuracy and consistency of ^89^Zr-trastuzumab.

### ULD-CT

To evaluate the robustness of the PET/CT coregistration framework when applied to ULD-CT, 7 patients with a significant visual mismatch between their LD-CT and ULD-CT scans were selected from a cohort of 27 patients scanned with ^18^F-FDG for both LD-CT and ULD-CT.

**FIGURE 5.**
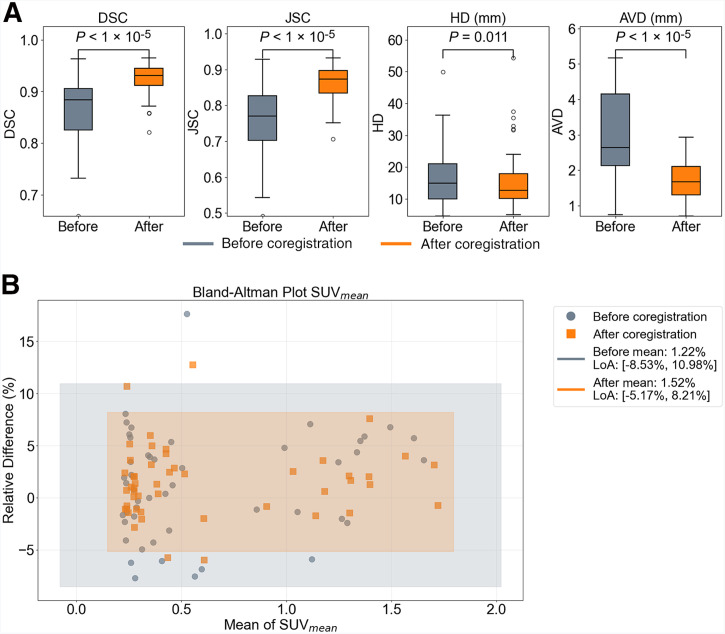
(A) Segmentation metrics (DSC, JSC, HD, AVD) between LD-CT and ULD-CT before and after coregistration. (B) Bland–Altman plot of SUV_mean_ values between PET reconstructions using ULD-CT and LD-CT (gray) and between rULD-CT and rLD-CT (orange).

As shown in Figure 5A, the coregistration framework improved the alignment between LD-CT and ULD-CT, as evidenced by enhancements across all metrics. After coregistration, DSC and JSC increased, indicating better overlap between segmentations, whereas HD and AVD decreased, reflecting improved boundary accuracy and reduced spatial discrepancies. These improvements were statistically significant (e.g., *P* < 1 × 10^−5^ for DSC, JSC, and AVD; *P* = 0.011 for HD).

The Bland–Altman plot (Fig. 5B) revealed a strong agreement between PET images reconstructed using rLD-CT and rULD-CT. The mean relative difference was 1.52%, with the LoA ranging from −5.17% to 8.21%. Notably, the coregistered datasets showed a narrower LoA than that calculated between the original LD-CT and ULD-CT.

## DISCUSSION

This study evaluated a deep-learning coregistration framework for mitigating motion-induced artifacts in PET/CT alignment on a LAFOV PET/CT. The framework improved PET/CT alignment across tracers, body regions, reduced PET counts, and CT scanning protocols, demonstrating robust performance. Misalignments between NAC-PET and CT were observed in multiple regions, and applying the proposed framework improved the alignment between PET and CT images, potentially aiding ROI selection (5). In addition, after applying this framework, rPET images showed a marked reduction in banana-shaped artifacts, leading to more accurate tracer activity recovery in the liver dome. This resulted in a more uniform and anatomically consistent tracer distribution, which is important for more accurate quantification and diagnostic interpretation in regions affected by respiratory motion (15). Importantly, the entire framework is fully automated and can be performed within a few minutes per dataset, enabling efficient integration into imaging workflows.

The framework demonstrated remarkable robustness when applied to reduced-count scenarios, where count statistics were progressively reduced to 50%, 25% and 12.5% of the original data. Despite the increased noise in these lower statistic images, the framework maintained high anatomic alignment, showing that it can be used in lower-dose or shorter scan-time protocols. This is particularly evident for ^18^F-MC225 and ^15^O-H_2_O, which consistently achieved the highest DSC and JSC and lowest HD and AVD across all count levels, with minimal degradation in performance, even at 12.5% counts. It is worth noting that ^15^O-H_2_O showed slightly poorer performance, which may be because 2 of 5 patients were scanned with a lower dose for a shorter duration (200 MBq and 370 s versus 500 MBq and 610 s). The lower activity and shorter acquisition duration for ^15^O-H_2_O caused higher noise in PET images, which could have impacted the framework’s performance. ^89^Zr-trastuzumab exhibited larger variability, particularly at the 12.5% count level. At this reduced dose, the LC-rCT became more distinct from the rCT, and all performance metrics showed increased dispersion. This is likely attributable to the inherently lower signal-to-noise ratio of ^89^Zr-trastuzumab on day 4 after injection (31), which becomes more pronounced as the PET counts are reduced to one eighth of the original, corresponding to an effective injected activity of 4.64 MBq.

A similar trend was observed in the quantitative analysis (Fig. 4). SUV_mean_ showed good agreement between reduced-count and original-count groups across all tracers. However, ^18^F-MC225 showed slightly worse performance compared with ^15^O-H_2_O because of its heterogeneous uptake across multiple region segments, which increases the sensitivity of PET quantification to errors in CT segmentation. For ^89^Zr-trastuzumab, 3 notable outliers were observed at the 12.5% count level, with relative differences in SUV_mean_ ranging from 10% to 15%. Notably, previous studies have also reported a bias of up to 10% in SUV_mean_ when comparing full-scan duration to half-scan duration PET data, even when using the same CT for attenuation correction (26). This suggests that the observed bias in the low-dose scenario is consistent with known variability in PET quantification at reduced counts scenarios. These findings indicate that, even if the proposed framework is robust, some degree of variability in PET quantification is expected, particularly for tracers like ^89^Zr-trastuzumab, where lower signal-to-noise ratios at reduced counts can magnify this effect.

This study shows that the proposed framework effectively mitigates the impact of noise in ULD-CT by leveraging NAC-PET data as a reference, leading to reliable and comparable results to LD-CT. Improvements in DSC, JSC, HD, and AVD confirm that the framework reduces misalignment between LD-CT and ULD-CT, even in cases with significant initial discrepancies. The enhanced agreement in PET quantification, as indicated by the narrower LoA and comparable mean relative differences, further underscores the robustness of the framework even when ULD-CT protocols are used.

Despite these promising results, our investigation had some limitations. First, in breath-hold scans, the framework does not fully account for changes in lung density (30). This limitation results in discrepancies between the deformed and original μ maps, particularly in the middle of the lungs, where a mean relative difference of at least 10% is expected (32), although it was not observed in Figure 3. In addition, observed differences in CT and PET segmentations may not be solely due to CT–PET coregistration errors. They could also reflect variability in the segmentation process, which can be affected by image quality and anatomic complexity. To minimize this effect, only large anatomic segments were analyzed, as they are generally less prone to segmentation inaccuracies than smaller or more irregular regions.

To enhance the utility of this framework, several improvements should be considered. These include incorporating rigid registration for regions such as the skull to better address alignment in rigid-body structures. Additionally, federated learning may offer a practical way to further improve performance by enabling access to multicenter data while preserving patient privacy (33). Furthermore, the use of tracers with distinct distribution in this study supports the framework’s generalizability. However, since ^15^O-H_2_O and ^18^F-MC225 are often acquired dynamically for kinetic analysis (24,25), further evaluation is needed to assess applicability in dynamic PET studies. Importantly, multiple studies have reported varying lesion-level results when applying the same PET/CT coregistration framework (16,34). One study demonstrated improved lesion-to-background ratios near the diaphragm (16). Another study reported lower performance of the registration framework relative to data-driven gating CT (34). In that study, the performance was likely influenced by variations in scanner performance characteristics, which were from the Siemens Biograph Vision system used for network training, as well as by differences in image reconstruction and correction methods. These factors can make fine-scale image registration more challenging, particularly for small structures. Nevertheless, the registration framework improved quantification within the lesion population. Further investigations are important to assess the framework’s generalizability across different clinical sites and more diverse patient groups (e.g., children, pregnant women).

## CONCLUSION

A deep-learning coregistration framework reduced motion-induced misalignments between PET and CT across a range of distinct tracers, scanning conditions, PET count statistics, and CT scanning protocols for LAFOV PET/CT.

## DISCLOSURE

Support for this work was provided by a partnership of University Medical Center Groningen and Siemens Healthineers for building future of health (C00240441 PUSH project agreements 33 and 41). Zekai Li is supported by a Joint Scholarship from the China Scholarship Council and the University of Groningen (202210100011). Charalampos Tsoumpas is funded by the NWO Talent Program Vici (21609). Charalampos Tsoumpas, Jean-Paul de Vries, Andor Glaudemans, and Riemer Slart have received funding for research from Siemens Healthineers. Paul Schleyer, Maurizio Conti, and Joshua Schaefferkoetter are full-time employees of Siemens Medical Solutions, USA. No other potential conflict of interest relevant to this article was reported.

## Notes

KEY POINTS **QUESTION:** Can a deep-learning PET/CT coregistration framework reduce motion-induced misalignments between PET and CT across a range of tracers and scanning protocols for LAFOV PET/CT? **PERTINENT FINDINGS:** In this study involving 63 PET scans acquired with four different tracers, the deep-learning PET/CT coregistration framework demonstrated robust coregistration performance using low-count PET images and ULD-CT across all tracers, as indicated by high DSC and JSC and low HD and AVD. The SUV_mean_ remained consistent across conditions. **IMPLICATIONS FOR PATIENT CARE:** Improved PET and CT alignment across a range of distinct distributed tracers, reduced dose PET and CT protocols without compromising quantification accuracy.
